# A comparison of the prevalence of sexually transmitted infections among circumcised and uncircumcised adult males in Rustenburg, South Africa: a cross-sectional study

**DOI:** 10.1186/s12889-021-10509-1

**Published:** 2021-04-06

**Authors:** Blanchard Mbay Iyemosolo, Tawanda Chivese, Tonya M. Esterhuizen

**Affiliations:** 1 Department of Global Health, Division of Epidemiology and Biostatistics, Faculty of Medicine and Health Sciences, University of Stellenbosch, Cape Town, South Africa; 2 Department of Population Medicine, College of Medicine, QU Health, Qatar University, Doha, Qatar

**Keywords:** Male circumcision, Traditional circumcision, High-risk population, Sexually transmitted infections (STIs), South Africa

## Abstract

**Background:**

South Africa has a persistent burden of sexually transmitted infections (STIs). Male circumcision has been shown to be effective in preventing HIV and STIs, but data are scarce on the protective effect of circumcision in high-risk populations such as migrant miners. The objective of this study was to assess the effect of medical and traditional circumcision on the prevalence of STIs after adjusting for other risk factors in Rustenburg, a mining town in North West Province, South Africa.

**Methods:**

This cross-sectional study used baseline data collected from a cohort study. Adult males in a mining town were assessed for STIs (gonorrhea, chlamydia, and trichomoniasis) using syndromic assessment. Data on circumcision status and other risk factors for STI syndromes were collected using an interviewer-administered questionnaire. The following symptoms were assessed; penile discharge, painful urination, dyspareunia or penile sores. These symptoms indicate sexually transmitted infection in general since laboratory tests were not performed. Multivariable log binomial regression was used to assess the independent effect of circumcision on STI presence after adjusting for confounders.

**Results:**

A total of 339 participants with a median age of 25 years (IQR 22–29) were included in the study, of whom 116 (34.2%) were circumcised. The overall STIs prevalence was 27.4% (95% CI 22.8 to 32.6%) and was lower in the circumcised participants compared with those who were uncircumcised (15.5% vs 33.6%, respectively, *p* < 0.001). Both medical (OR 0.57, 95% CI 0.34–0.95, *p* = 0.030) and traditional circumcision (OR 0.34, 95% CI 0.13–0.86, *p* = 0.022) were strongly associated with a lower risk of STIs after adjustment for employment and condom use.

**Conclusion:**

In this high-risk population in a mining town in South Africa, with a relatively high prevalence of STIs, and where one third of males are circumcised, both medical and traditional circumcision appear to be protective against STIs.

## Background

The global burden of sexually transmitted infections (STIs) remains high [1]. Worldwide, more than a million people acquire sexually transmitted infections every day, 499 million new cases of treatable STIs (gonorrhea, chlamydia, syphilis, and trichomoniasis) arise each year, and 536 million people are estimated to be living with incurable herpes simplex virus type 2 (HSV-2) [2]. In South Africa, several surveys have shown that the prevalence of STIs is considerable in childbearing age groups. During the period 2016–17, one study estimated the prevalence of chlamydia and herpes simplex virus type 2 to be 5 and 17%, respectively [3]. A considerable prevalence of STIs has also been described in young men and women, with the same study reporting a prevalence of 2% for gonorrhea, and 5% for trichomoniasis in young man and women aged between 15 and 24 years old in rural KwaZulu Natal [3]. There is a need to both promote and evaluate existing interventions to improve the effectiveness of prevention of STIs.

STIs have varying consequences on sexual and reproductive health. Syphilis in pregnancy leads to an estimated 305,000 adult and new-born deaths and leaves 215,000 babies at high risk of losing their life from prematurity, low birth weight or congenital disease each year in the world [2]. Gonorrhea and chlamydia are risk factors for infertility, and untreated genital infection may be the cause of up to 85% of sterility of women seeking infertility treatment [2]. As a result of awareness of the persistent problem of STIs, as well as the effects on health, the World Health Organization (WHO) Global Health Sector strategy on STI 2016–2021 [4] has outlined the goals and targets for global STIs prevention and control. Among these targets is to collect information on STI prevalence and incidence across representative populations [4]. STI diagnosis and management is usually through syndromic management. Syndromic management of STIs uses clinical algorithms such as decision trees for commonly presenting signs and symptoms. These are also used in case management as the symptoms selected are reasonably consistent and easy to recognize, and therefore the algorithm provides treatment for the commonest biological causes of the syndrome [5]. Although the etiological diagnosis using laboratory confirmation is considered as the gold standard for diagnosis and management of sexually transmitted infections [6], this strategy may delay the patient’s treatment and follow-up.

In South Africa, the government is promoting voluntary male circumcision and condom use [7] and has sentinel surveillance in place across the nine provinces [8]. However, the burden of STIs remains high and this may require combining biological and behavioral interventions. Further, the use of condoms has remained low in South Africa despite efforts by the government to make condoms accessible to all [9].

Male circumcision is one of the oldest and most common surgical procedures worldwide and is accepted for many reasons including religious, cultural, social and medical [7]. Circumcision decreases the entry of pathogens through abrasions in the thinly keratinized inner mucosal surface of the foreskin and eliminates the moist environment under the foreskin, which may favor pathogen persistence and reproduction [4]. From an efficacy point of view, there is conclusive evidence that male circumcision reduces the risk of HIV and STI infection [10–12], and the WHO now recommends voluntary medical male circumcision (VMMC) as one of the key HIV and STI prevention strategies [13]. Circumcision does not only protect the male partner, but evidence shows that the female partner is also protected. This is supported by data from a systematic review of 57 observational studies which showed that females were at a decreased risk of STIs when their male partners were medically circumcised [14]. There is also ample evidence that circumcision reduces risk of HIV and STIs in men-who-have-sex with men (MSM), reducing risk of HIV infection by up to 23%, as shown by data from a meta-analysis of 62 observational studies [15].

Although data from clinical trials and several observational studies support the efficacy of male circumcision in reducing the risk of STDs, a few questions remain either unanswered or requiring more research. One of these questions is whether male circumcision remains efficacious in settings where high-risk behaviors are prevalent such as the mining towns in South Africa. Mining towns in South Africa are characterized by high-risk sexual behavior, as most miners are “mobile men with money”, migrant workers who tend to stay in the single sex hostels, a remnant of the apartheid system [16–18]. These towns also tend to have large populations of female sex workers [16], attracted by the “mobile men with money”, the migrant miners. STIs tend to be diagnosed late in women and female sex workers may act as reservoirs of infection and increase the risk of incident cases and re-infection [19]. Further, one of the unintended effects of widespread educational campaigns about the benefits male circumcision is behavioral risk compensation, whereby circumcised men expose themselves more, in the belief that they will not get STIs [20]. Behavioral risk compensation is not limited to men only as women may change their behavior in the belief that VMMC protects them. In one study in South Africa, women who were aware of the benefits believed that VMMC reduced the need to worry about HIV and were less likely to use condoms with circumcised men [21]. Furthermore, even when the men are circumcised, some resume sexual activity before the 6 weeks post circumcision wound healing period and this increases the risk of HIV [20]. It is still not clear whether early resumption of sexual activity increases risk of STIs. How these factors affect the risk of STIs in a setting where high-risk sexual activity is prevalent such as migrant mining communities found in certain parts of South Africa is not known [22]. This is coupled with the fact that many men are circumcised using traditional ways as many communities practice circumcision as a rite of passage to manhood [23]. Available data suggest that traditional circumcision may not offer optimum protection against the risk of STIs through a lack of health education, incomplete foreskin removal and higher risky sexual behaviors such as multiple partners and lower condom use [23].

This study compares the prevalence of STI syndrome between men who are circumcised and those who are not, in an observational setting, where high-risk sexual behaviors are prevalent. Further, we compared the prevalence of STI syndrome between males who were traditionally circumcised and those medically circumcised. Lastly, we assessed the effect of circumcision on the risk of STIs, after adjusting for established risk factors for STI syndrome.

## Methods

### Study design and eligibility and recruitment

We used baseline cross-sectional data on men collected from a previous study, therefore, this study is a secondary data analysis. The full details of the parent study are described elsewhere [24], and brief details will be described in this study. The parent study investigated HIV incidence and predictors of inconsistent condom use among adult men enrolled into an HIV vaccine preparedness study in Rustenburg South Africa and was conducted between May 2012 and Jun 2015. In the parent study, men aged 18–49 years of age were recruited from urban and rural area of Rustenburg by the research staff. The research staff used awareness campaigns and community meetings to recruit men into the HIV vaccine preparedness study. Further, the research staff approached men in various settings such as the central business district, townships, clinics, shopping centres, taxi ranks, taverns and car wash stations. Snowball sampling was then used to recruit friends and acquaintances of the enrolled men into the study. Men who consented to participate were tested for HIV and completed a screening questionnaire for high-risk sexual behaviour. Participants reporting previous STI diagnosis, multiple sexual partners, having sexual intercourse with a man (MSM), having a new sexual partner or having sexual intercourse with a partner who was known to have HIV positive were classified as “high-risk”. Only participants who were HIV negative were enrolled into the parent study. The same eligibility criteria were used for this sub-study except that men who were MSM were not included in this study.

### Setting

Rustenburg city is a mining town in the Bojanala Platinum District in the North West province, 173 km from Johannesburg. The Tshwana ethnicity and other diverse internal and external migrants are the predominant populations in this community [24]**.** The migration population is due to the mining industry which attracts many people seeking employment. Further, most of the migrant mine workers are male and are housed in single sex male hostels, and this is associated with high numbers of female sex workers [25]. This may increase the prevalence of STIs in this population [26].

### Sample size and power

The baseline data on 339 heterosexual men collected over 6 months were available for analysis. Post –hoc power calculation based on the prevalence of STIs being 30%, prevalence of all types of circumcision being 34%, and among those circumcised, 15% having STIs while among the uncircumcised group a 30% prevalence of STIs, yielded 84.8% power to detect a difference.

### Data collection

Data were collected using a validated interviewer-administered questionnaire, published elsewhere [24]. Data collected included socio-demographic data, risky sexual behaviour and circumcision status and assessment of STIs. Inconsistent condom use was categorized as never, frequently, or sometimes using condoms with partners in the last 3 months, while consistent condom use was always using a condom with all partners.

### Assessment of circumcision

Using the interviewer-administered questionnaire, participants were asked whether they were circumcised and if so, whether they were circumcised traditionally or medically. There was no distinction between cultural and traditional circumcision.

### Assessment of STIs

Assessment of sexually transmitted infections was done in the parent study using syndromic assessment. This included a brief physical examination and assessment of vital signs, weight, height, examination external genital, and rectal examinations for relevant symptoms [24]. Clinicians employed by the study conducted the syndromic assessment, which entailed assessment for symptoms of STIs such as genital area, presence of genital ulcers, and any discharge. Because we assessed for symptoms of STIs only, without laboratory confirmation, the study outcome was the prevalence of STI syndrome.

### Data analysis

All analyses were performed using Stata version 15 (Stata Corp (2017) Stata Statistical Software: Release 15. College Station, TX: Stata Corp LLC). Numerical data were presented using mean and standard deviation (SD) when variables were normally distributed, otherwise median and inter-quarter ranges (IQR) were reported. Frequencies and percentages were used to summarize categorical variables.

Demographic data were compared between the circumcision categories using Kruskal Wallis tests, chi-squared or Fisher’s exact tests. The prevalence of STI syndrome overall was presented with 95% confidence intervals (95%CI), and compared between men who were not circumcised, those who were traditionally circumcised and those who were medically circumcised. The association between risk factors including circumcision and STI syndrome prevalence was assessed initially using univariate log binomial regression analysis and crude relative risks and 95% confidence intervals reported. Circumcision as well as the other risk factors which were found to show an association with STI syndrome at <= 0.2 level of significance were included in the predictive multivariable log binomial regression model. Backward selection based on likelihood ratios was used to arrive at a final model. The magnitude of the association between identified factors and outcomes was summarized using adjusted relative risks and 95% confidence intervals.

### Ethics considerations

The study was approved by the Health Research Ethics Committee at Stellenbosch University, (Reference No: S19/07/134), with a waiver of informed consent, as no new data were collected from participants. Permission to use the data was obtained from the Arum Institute.

## Results

Table 1 shows the demographics of the participants by circumcision status. Baseline data on 339 participants were included in the study. In total 116 (34.2%) of the participants were circumcised, by either medical or traditional means. The majority (68%) were medically circumcised while the remainder were traditionally circumcised. The median age of the sample was 25 years, with an inter-quartile range from 22 to 29 years. Median age was very similar in those who were circumcised medically (median 25 years, IQR 23–29 years) and culturally (median 27 years, IQR 23–32 years), with the non-circumcised participants (median 25 years, IQR 22–28 years). The mean total years of schooling was 12 years with a standard deviation of 2 years. Most of the participants were Black, married, but lived without a partner. Demographics did not vary by circumcision status (Table 1).

**Table 1:** Demographics of participants by circumcision status

		circumcision type				*p*-value
		Not circumcised	Medically circumcised	Traditionally circumcised	Total	
Enrolment Age (years)	Median (IQR)	25 (22–28)	25 (23–29)	27 (23–32)	25 (22–29)	0.147
Age of sexual debut (years)	Median (IQR)	17 (16–18)	16 (15–18)	17 (16–18)	17 (15–18)	0.066
Total years of schooling (years)	Median (IQR)	12 (12–14)	12 (12–13)	12 (10–13)	12 (12–13)	0.280
Race n (column %)	Black	220 (98.7)	79 (100.0)	37 (100.0)	336 (99.1)	0.813
	White	1 (0.4)	0 (0.0)	0 (0.0)	1 (0.3)	
	Other	2 (0.9)	0 (0.0)	0 (0.0)	2 (0.6)	
Marital status n (column %)	Single	215 (96.4)	72 (91.1)	34 (91.9)	321 (94.7)	0.074
	Divorced	0 (0.0)	2 (2.5)	0 (0.0)	2 (0.6)	
	Married	8 (3.6%	5 (6.3)	3(8.1)	16 (4.7)	
Does your partner live with you n (column %)	No	168 (75.3)	61 (77.2)	30 (81.1)	259 (76.4)	0.780
	Yes	36 (16.1)	13 (16.5)	6 1 (6.2)	55 (16.2)	
	Not applicable/ no partner	19 (8.5)	5 (6.3)	1 (2.7)	25 (7.4)	
Employment n (column %)	Unemployed	110 (49.3%)	37 (46.8%)	19 (51.4%)	166 (49.0)	0.701
	Self employed	16 (7.2%)	6 (7.6%)	1 (2.7%)	23 (6.8)	
	Student	16 (7.2%)	9 (11.4%)	1 (2.7%)	26 (7.7)	
	Employed	79 (35.4)	27 (34.2)	16 (43.2)	122 (36.0)	
	Other	2 (0.9)	0 (0.0)	0 (0.0)	2 (0.6)	

The prevalence of STIs overall was 27.4% (93/339) (95% CI 22.8 to 32.6%). The prevalence of STIs was higher in uncircumcised males, compared to those circumcised (33.6% vs 15.5%, respectively). Further, the prevalence of STI syndrome in participants who were medically circumcised was 17.7% versus 10.8% in those who were traditionally circumcised.

Table 2 shows univariate associations between risk factors and STI syndrome prevalence. Circumcised males, both medically circumcised and traditionally circumcised, were less likely to develop STI syndrome than uncircumcised men. Employed men were more likely to have STI syndrome than unemployed men (Table 2). Always using condoms was significantly protective for STI syndrome. The enrolment age and years of schooling were weakly positively associated with STI syndrome.

**Table 2:** Univariate association between circumcision, other risk factors, and STI presence

Factor	Levels	STI Presence		Crude RR (95% CI)	*P*-value
		Present (*n* = 93)	Absent (*n* = 246)		
Circumcision	No	75 (33.6%)	148 (66.4%)	1 (reference)	
	Medically circumcised	14 (17.7%)	65 (82.3%)	0.527 (0.317–0.877)	0.014
	Traditionally circumcised	4 (10.8%)	33 (89.2%)	0.321 (0.125–0.826)	0.018
Employment	Unemployed	35 (21.1%)	131 (78.9%)	1 (reference)	
	Self employed	8 (34.8%)	15 (65.2%)	1.650 (0.877–3.105)	0.121
	Student	5 (19.2%)	21 (80.8%)	0.912 (0.393–2.115)	0.830
	Employed	44 (36.1%)	78 (63.9%)	1.711 (1.173–2.495)	0.005
	Other	1 (50.0%)	1 (50.0%)	2.371 (0.575–9.780)	0.232
Does your partner live with you?	No	66 (25.5%)	193 (74.5%)	1 (reference)	
	Yes	20 (36.4%)	35 (63.6%)	1.824 (0.451–7.376)	0.399
	Not applicable/no partner	7 (28.0%)	18 (72.0%)	1.099 (0.567–2.131)	0.780
During the last month, on average, how often have you had a drink Containing alcohol?	none	23 (25%)	69 (75%)	1(reference)	
	1–3 times	39 (27.5%)	103 (72.5%)	1.099 (0.705–1.712)	0.678
	Weekly	27 (28.4%)	68 (71.6%)	1.137 (0.706–1.831)	0.598
	Daily	4 (40%)	6 (60%)	1.6 (0.692–3.697)	0.271
During the last month, how often were you drunk/drinking alcohol before sex?	never	128 (27.7%)	49 (72.3%)	1 (reference)	
	sometimes	91 (28.3%)	36 (71.7%)	1.024 (0.711–1.475)	0.899
	frequently	18 (18.2%)	4 (81.8%)	0.657 (0.262–1.644)	0.369
	always	9 (30.8%)	4 (69.2%)	1.111 (0.475–2.599)	0.807
Frequency of use of condom with female partner(s)	Never	18 (38.3%)	29 (61.7%)	1 (reference)	
	Sometimes	43 (32.6%)	89 (67.4%)	0.851 (0.549–1.318)	0.469
	Frequently	16 (22.2%)	56 (77.8%)	0.580 (0.330–1.020)	0.059
	Always	13 (15.3%)	72 (84.7%)	0.399 (0.215–0.741)	0.004
Enrolment Age in years: median (IQR)		26 (23–30)	25 (22–28)	1.021 (0.993–1.050)	0.144
Age group	20–23	39 (25.5%)	114 (74.5%)	1 (reference)	
	24–29	27 (24.8%)	82 (75.2%)	0.972 (0.635–1.486)	0.895
	30–34	16 (40%)	24 (60%)	1.569 (0.984–2.501)	0.058
	35–39	7 (26.9%)	19 (73.1%)	1.056 (0.530–2.103)	0.876
	> = 40	4 (36.4%)	7 (63.6%)	1.427 (0.624–3.263)	0.400
Age of sexual debut, in years: median (IQR)		17 (16–18)	17 (15–18)	0.983 (0.933–1.035)	0.514
Years of schooling: median (IQR)		12 (11–13)	12 (11–14)	1.069 (0.974–1.173)	0.157

The five covariates (circumcision, employment, condom use, age and years of schooling) were entered into a multiple log binomial regression model. Using backward selection based on likelihood ratios, within three steps the final model containing circumcision, employment, and frequency of condom use remained. Circumcision remained strongly protective for STI syndrome (Adjusted RR for medically circumcised 0.565 and 0.339 for traditionally circumcised, *p* = 0.030 and 0.022 respectively), after adjusting for employment, and condom use, as shown in Table 3. Frequent condom use was still significantly protective even after adjustment for circumcision. Employed men were 1.64 times more likely to have STI syndrome after adjustment for circumcision and condom use (Table 3).

**Table 3:** Multiple log binomial regression model for risk factors for STIs

Factor	*p* value	Adjusted Relative Risk	Lower 95% CI	Upper 95% CI
Circumcised (medically vs no)	0.030	0.565	0.337	0.947
Circumcised (traditionally vs no)	0.022	0.339	0.134	0.857
Employment				
Self-employed vs unemployed	0.128	1.592	0.875	2.898
Student vs unemployed	0.614	0.782	0.302	2.031
Employed vs unemployed	0.009	1.639	1.1339	2.370
Other vs unemployed	0.530	1.665	0.339	8.172
Frequent use of condom with female partner(s)				
Sometimes vs never	0.598	0.893	0.586	1.356
Frequently vs never	0.137	0.660	0.381	1.141
Always vs never	0.015	0.480	0.266	0.866

## Discussion

In this study, we found a high prevalence of 27.4% for STI syndrome overall, and that uncircumcised males had double the prevalence compared to circumcised males. We also found that the prevalence of STI syndrome was higher in individuals who were medically circumcised, compared to those who were traditionally circumcised. Finally, we found that both medical and traditional circumcision were independently associated with a protective effect against the risk of STI syndrome.

Several RCTs [11, 12, 27–29] and several meta-analyses in heterosexual males [30], in MSM [30] and in women [31] have demonstrated that male circumcision is protective against HIV. We found that male circumcision is beneficial against STI syndrome, a finding that is in line with data from the RCTs, despite the differences in the outcome and study design. Syndromic STIs increase risk of HIV acquisition, therefore our findings show an added benefit of male circumcision in that it may reduce risk of both syndromic STIs and HIV, in high risk settings. Although data on the proportion of men with symptoms of STIs who have STIs confirmed by laboratory testing is sparse, one study in South Africa showed that up to 90% of men with urethral discharge syndrome have at least one confirmed STI [32]. This shows that syndromic STI assessment is a sensitive measure for STIs. Our study adds to the body of knowledge by contributing findings on the protective effect of male circumcision in heterosexual men in a high-risk mining town environment, as well as data on the prevalence of STI syndrome in traditionally circumcised men. However, the high prevalence of STI syndrome in our study setting suggests a need for more interventions in the mining towns. These interventions could include provision of more medical circumcision services and more health education to reduce risky sexual behaviors and to encourage the miners to be circumcised. There is also need for research to investigate the drivers for the high prevalence of STIs, as well as the acceptability of medical circumcision in the miners, as they usually come from diverse backgrounds.

We found a lower prevalence of STI syndrome in men who were traditionally circumcised compared to both the medically circumcised and those not circumcised. It is not clear why the men who were medically circumcised had a higher prevalence of STI syndrome, compared to those traditionally circumcised, in our study. Bigger robust studies are needed to assess this finding. While our finding that traditional circumcision may have a protective effect against STI syndrome is a positive finding, the findings need to be interpreted with caution. Traditional circumcision has several issues which need to be dealt with, if it is going to be a safe and efficacious method of preventing STIs and HIV. Data shows that there is a higher risk of complications when traditional circumcision is used, compared to medical circumcision [33]. A serious concern against traditional circumcision in South Africa is the high number of circumcision-related deaths that are reported every year [34]. Several interventions have been assessed, including the training of traditional “surgeons” in aseptic technique and the need to refer men who develop complications [34]. However, more effective interventions are still needed to reduce the number of these largely preventable deaths of initiates [34].

Our data shows a huge disparity in consistent condom use between participants with STI syndrome and those without. The two interventions, condom use and male circumcision, if combined, may provide a greater protective effect against both STIs and HIV. However, in South Africa, condom use has remained suboptimal [35] while the prevalence of male circumcision varies from a low of 25% to a high of 75% between districts [36]. The results of our study suggest a strong need to increase coverage of voluntary male circumcision. Several service-delivery interventions have been found to increase the uptake of voluntary male circumcision. These interventions include the provision of mobile circumcision services, home based HIV testing and referral services and comprehensive sex education [37]. Data also suggests that these interventions are not only perceived as acceptable but are cost effective and feasible in low resource setting [37]. More research is needed on interventions to improve coverage of voluntary male circumcision, as part of the package of interventions for the prevention of HIV and STIs.

A limitation of this study is the cross-sectional design which limits our ability to explain the cause-and-effect relationship between the two circumcision methods and prevention of STIs. We can merely state an association that is apparent in the data. Another limitation is that both circumcision and risky sexual behavior were determined by self- reports of participants. Risky sexual behaviors might have been under-reported, while circumcision could have been over reported as it is traditionally desirable in most cultures. Further, we did not assess whether circumcision was done culturally or just by a traditional practitioner and therefore we are not able to assess the effect of these two practices. The prevalence of STI could have been underestimated in this study because the STIs that are not syndromic were not assessed. These STIs include HIV, the Human Papilloma Virus (HPV), trichomoniasis and Syphilis. However, all the participants were tested for HIV at the beginning of the parent study and the results are reported elsewhere [24]. The assessment of circumcision did not include an examination of whether their circumcision was partial or complete, since in some cases of traditional male circumcision, the removal of the foreskin is partial and in others it is complete, which is protective. We therefore were unable to assess the effect of partial circumcision.

## Conclusion

Both medical and traditional circumcision are associated with lower risk of sexually transmitted infections in a population with a relatively high STI syndrome prevalence and where one-third of males are circumcised. Further evidence from robust randomized controlled trials may be required to assess the efficacy of traditional male circumcision as a prevention tool against STIs in this population.

## Data Availability

This study is a secondary analysis of the IAVI Protocol B study reported at 10.1371/journal.pone.0214786. Permission to use these data was obtained from the Regional Chief Operating Officer (Research) of The Aurum Institute on the 26th of November 2020. The datasets used and/or analyzed during the current study are available from the corresponding author on reasonable request.

## Abbreviations

STI: Sexually transmitted infection
HIV: Human immunodeficiency virus
HSV2: Herpes simplex virus 2
MSM: Men who have sex with men
SPSS: Statistical package for social science
AOR: Adjusted odds ratio
CI: Confidence intervals
IRQ: Inter-quarter range
VMMC: Voluntary medical male circumcision
